# Stump Appendicitis: A 25-Year Review of Pathophysiology, Diagnosis, and Management (2000-2025)

**DOI:** 10.7759/cureus.107297

**Published:** 2026-04-18

**Authors:** Anupam Gupta

**Affiliations:** 1 Surgery, Bangalore Medical College, Bangalore, IND; 2 Surgery, Jackson Memorial Hospital, Miami, USA

**Keywords:** completion appendectomy, laparoscopic appendectomy, recurrent appendicitis, residual appendiceal stump, stump appendicitis

## Abstract

Acute appendicitis is one of the most common causes of right lower quadrant (RLQ) abdominal pain, and appendectomy remains the standard treatment. However, residual appendiceal tissue may persist after surgery and subsequently become inflamed, resulting in stump appendicitis, a rare but clinically significant condition. This review summarizes the current literature on stump appendicitis from 2000 to 2025, focusing on pathophysiology, clinical presentation, diagnostic modalities, and management strategies. A comprehensive search of PubMed, Google Scholar, and Cochrane databases was performed, and relevant English-language studies were analyzed. Reported cases span a wide age range, with variable intervals between index appendectomy and presentation. Most patients present with RLQ pain and imaging findings consistent with appendiceal inflammation, although diagnosis is often delayed due to low clinical suspicion. Computed tomography remains the diagnostic modality of choice. Management is typically completion appendectomy; however, selected uncomplicated cases may be initially managed conservatively with antibiotics, with or without interval surgery. Complicated cases may require percutaneous drainage or more extensive resection. Stump appendicitis remains under-recognized, and greater awareness, standardized reporting, and prospective studies are needed to improve diagnosis and guide management.

## Introduction and background

Acute appendicitis remains one of the most common surgical emergencies presenting with right lower quadrant (RLQ) pain [1]. The lifetime risk of developing acute appendicitis is approximately 7%. In contrast, development of stump appendicitis -- inflammation of residual appendiceal tissue after appendectomy -- is much rarer, with historical estimates of incidence at about 1 in 50,000 [1,2].

Patients present with RLQ pain, nausea/vomiting, and imaging features of an inflamed appendix. The standard treatment is appendectomy, increasingly performed laparoscopically [3]. In theory, after identification of the inflamed appendix, ligation of the meso-appendix and removal flush to the cecum should eliminate future risk. However, due to surgical and anatomical factors (inflamed base, retrocecal position, and limited vision in laparoscopy), some appendiceal tissue may remain. Over time, that residue may become inflamed, causing stump appendicitis, potentially with abscess, perforation, or even neoplastic change [4]. Because of the prior appendectomy, clinicians may have a low index of suspicion for appendicitis, leading to delayed diagnosis and increased morbidity.

Despite increasing reports, the condition remains under-recognized and lacks formal guidelines. Previous reviews (for example, [5]) cover a limited number of cases. The aim of this review is to survey published literature (2000-2025), summarize clinical features, diagnostic modalities, and management strategies, and identify gaps in knowledge.

## Review

I performed a systematic literature search in accordance with the Preferred Reporting Items for Systematic Reviews and Meta-Analyses (PRISMA) guidelines. PubMed/MEDLINE, Google Scholar, and the Cochrane Library were searched for studies published from January 2000 to June 2025. The search strategy used Boolean combinations of keywords as follows: (“stump appendicitis” OR “appendiceal stump” OR “residual appendix inflammation” OR “recurrent appendicitis after appendectomy”) AND (“appendectomy” OR “post-appendectomy” OR “appendiceal remnant”).

I included English-language articles reporting stump appendicitis, including case reports, case series, and review articles. Articles published before 2000, non-English publications, cases involving congenital anomalies (e.g., malrotation), and duplicate datasets were excluded.

Titles and abstracts were screened for eligibility, followed by full-text review of relevant articles. Study selection and data extraction were performed by the author using predefined inclusion and exclusion criteria. From each eligible study, data were extracted on the year of publication, authors, number of patients, patient demographics (age and sex), clinical presentation, time interval since the index appendectomy, surgical approach to the initial appendectomy (open vs laparoscopic), diagnostic modality, and management outcomes.

A formal risk-of-bias assessment was not performed due to the predominance of case reports and case series, and this is acknowledged as a limitation.

**Figure 1:**
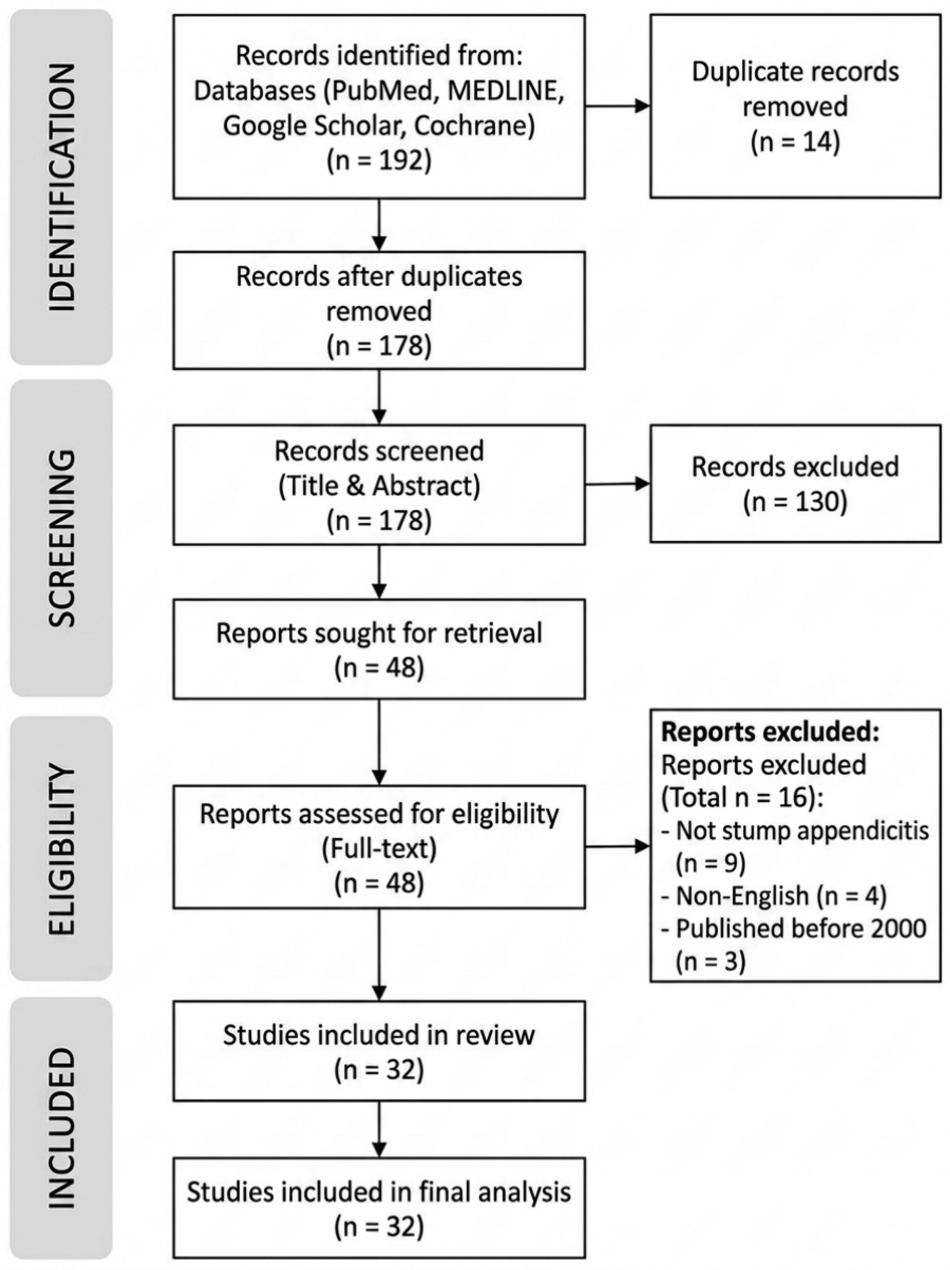
PRISMA flow diagram of study selection.

The study selection process is illustrated using a PRISMA flow diagram (Figure 1). This flow diagram illustrates the identification, screening, eligibility, and inclusion of studies on stump appendicitis published between 2000 and 2025. A total of 192 records were identified through database searching, and 26 additional records through other sources. After removal of duplicates, 178 records were screened, of which 130 were excluded. Forty-eight full-text articles were assessed for eligibility, with 16 excluded due to predefined criteria. A total of 32 studies were included in the final qualitative synthesis and analysis.

Data are summarized in Table 1.

**Table 1:** Summary of reported cases of stump appendicitis (2000-2025), including patient demographics, clinical presentation, time since index appendectomy, and initial surgical approach. RLQ = right lower quadrant; NR = not reported.

Study	Year	No of patients	Age (years)	Sex	Presentation	Time since appendectomy	Index appendectomy approach
Mangi & Berger [5]	2000	3	NR	NR	NR	NR	NR
Liang et al. [6]	2006	1	32	F	RLQ abdominal pain	5 months	NR
Al-Dabbagh et al. [7]	2009	1	41	M	RLQ pain	NR	NR
Awe et al. [8]	2013	1	25	F	NR	4.5 months	Laparoscopic
Hendahewa et al. [9]	2015	NR	NR	NR	NR	NR	NR
Rios et al. [10]	2015	2	NR	NR	RLQ pain	NR	NR
Dimaya & Sahu [11]	2016	39	NR	F	Peritonitis	1 year 2 months	NR
Dikicier et al. [12]	2018	5	19-45 (mean 32)	3M/2F	RLQ pain, peritonitis	4 years	NR
Rhyne et al. [13]	2018	1	45	F	RLQ pain	8 years	NR
Geraci et al. [14]	2019	NR	NR	NR	NR	NR	NR
Choi et al. [15]	2019	6	42.4	NR	NR	NR	NR
Baldwa et al. [16]	2020	1	66	F	Not reported	3 years	NR
Enzerra et al. [17]	2020	14	Mixed	Mixed	Acute abdominal pain	Varied	Mixed/NR
Mejri et al. [18]	2020	1	18	F	Acute RLQ pain, fever	~12 years	Laparoscopic
Erzurum et al. [19]	1997	1	13	M	Acute appendicitis	6 months	NR
Mizuta et al. [20]	2020	1	NR	NR	Acute abdomen	30 months	Laparoscopic
Hadrich et al. [21]	2021	1	NR	NR	Acute abdominal pain, obstruction	NR	NR
Castañeda et al. [22]	2021	1	NR	NR	Acute abdominal pain	NR	Laparoscopic
Basukala et al. [23]	2022	1	NR	NR	Perforation	1 year	Open
Keller et al. [24]	2022	1	42	M	RLQ pain, CT findings	NR	NR
Paudyal et al. [25]	2022	2	41, 23	M/F	RLQ pain	2 years; 1 month	NR
Mohammed et al. [26]	2023	1	26	M	RLQ pain, CT diagnosis	NR	NR
Alemayehu et al. [27]	2023	1	23	M	RLQ pain, perforation	7 months	Open
Yuen et al. [28]	2024	1	NR	NR	RLQ pain	NR	NR
Soh et al. [29]	2024	1	NR	NR	RLQ pain	NR	NR
Bastakoti et al. [30]	2024	1	61	M	RLQ pain	4 years	Open
Atri et al. [31]	2024	1	58	M	Abscess, RLQ pain	8 months	Laparoscopic
Nelluri & Gupta [32]	2024	1	59	F	Recurrent RLQ pain	NR	NR
Ochoa et al. [33]	2025	1	48	M	RLQ pain	NR	NR
Fasfoos et al. [34]	2025	1	17	M	Diffuse abdominal pain, fever	11 days	NR
Ismail et al. [35]	2009	1	2	M	RLQ pain	1 year	Open
O’Leary et al. [36]	2010	1	43	M	Abdominal pain	10 years	Open

Results

The reported cases demonstrated substantial heterogeneity in both patient characteristics and clinical course. Age at presentation ranged from adolescence to older adulthood, with reported cases spanning approximately 13 to 66 years, although some studies did not specify age. The interval between index appendectomy and presentation with stump appendicitis varied widely, ranging from as early as 11 days to as long as approximately 12 years. Both open and laparoscopic appendectomies were associated with subsequent stump appendicitis, although many earlier reports followed open surgery. The most common presenting symptom was RLQ abdominal pain, often accompanied by nausea, vomiting, fever, or signs of perforation or abscess formation in more advanced cases. Diagnostic delay was frequently described, largely because a prior appendectomy lowered clinical suspicion. Computed tomography was the imaging modality most commonly used to establish the diagnosis. Management was predominantly completion appendectomy, whereas complicated presentations sometimes required percutaneous drainage, ileocecectomy, or right hemicolectomy [5-35].

Pathophysiology

Stump appendicitis arises from inflammation of residual appendiceal tissue following incomplete resection during appendectomy. Several anatomical and surgical factors contribute to this condition. Retrocecal or subserosal positioning of the appendix may hinder complete visualization and removal, while significant inflammation during the initial surgery can obscure the appendiceal base and increase the likelihood of leaving a residual stump. In laparoscopic procedures, limited tactile feedback and restricted visualization may further contribute to incomplete resection [34-38].

Residual stump length has been identified as an important risk factor. A stump length greater than 3-5 mm has been suggested to increase the risk of stump appendicitis, as described by O’Leary et al. [36], while other reports have noted that stumps exceeding 1 cm are particularly associated with recurrent inflammation [4]. The underlying pathogenic mechanism parallels that of primary appendicitis and involves luminal obstruction due to fecalith formation or lymphoid hyperplasia, followed by increased intraluminal pressure, vascular compromise, and bacterial invasion.

Diagnosis

Patients with stump appendicitis typically present with clinical features similar to acute appendicitis, including RLQ pain, nausea, vomiting, and localized tenderness. Fever may also be present. However, a history of prior appendectomy often leads to reduced clinical suspicion, contributing to delays in diagnosis, as described in prior case series and reviews of stump appendicitis [4].

Imaging plays a critical role in diagnosis. Ultrasound may demonstrate a blind-ending tubular structure adjacent to the cecum, although its accuracy is operator-dependent and may be limited in detecting small residual stumps [17,36,38]. Contrast-enhanced computed tomography is considered the diagnostic modality of choice and may reveal an inflamed appendiceal stump, pericecal fat stranding, wall thickening, and associated complications such as abscess or fluid collection [17]. Reported stump lengths on CT imaging have reached up to 3.5 cm in some cases [36].

The differential diagnosis includes terminal ileitis, Meckel’s diverticulitis, ureteric colic, incisional or trocar-site hernia, epiploic appendagitis, and gynecological pathology in female patients. Prior appendectomy should not exclude appendiceal pathology from the differential diagnosis, as emphasized in radiologic and surgical reviews of stump appendicitis and RLQ pain [39-43].

Management

Completion appendectomy remains the standard treatment for stump appendicitis when an inflamed residual stump is identified. This can be performed via open or laparoscopic approaches, with laparoscopic surgery offering advantages such as reduced postoperative pain and shorter recovery times in appropriately selected patients [34-37].

In complicated cases involving abscess formation, management may include image-guided percutaneous drainage followed by interval completion appendectomy [37-39]. In more severe cases with extensive inflammation or cecal involvement, ileocecectomy or right hemicolectomy may be required [27]. Conservative management with antibiotics has been described in selected cases, particularly in patients with mild inflammation or those unfit for surgery [42-44]. However, data on long-term outcomes and recurrence rates remain limited.

Preventive strategies during the initial appendectomy are critical and include careful identification of the appendiceal base using anatomical landmarks such as the taenia coli, adequate ligation of the mesoappendix, and resection of the appendix as close to the cecum as possible. Documentation of stump length, ideally less than 3-5 mm, is recommended to reduce future risk [40-44].

**Figure 2:**
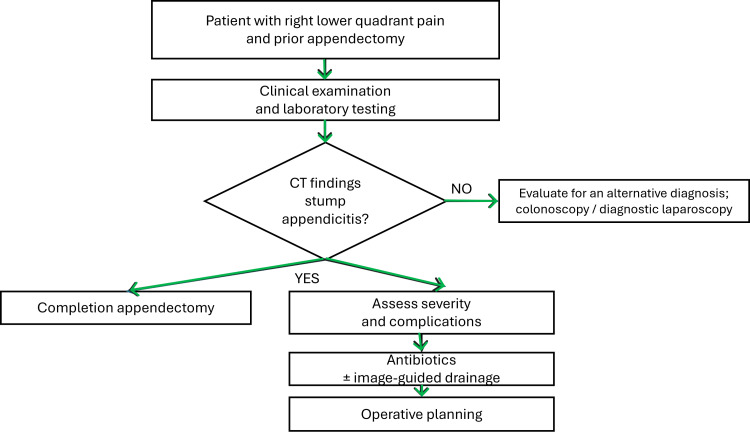
Proposed diagnostic and management algorithm for stump appendicitis. CT = computed tomography.

The algorithm for management suggested by us has been presented in Figure 2.

Discussion

This review highlights stump appendicitis as a rare but clinically significant complication following appendectomy. The wide variability in the interval between initial surgery and presentation underscores the need for long-term clinical awareness in patients presenting with RLQ pain despite a prior appendectomy. The clinical presentation closely mirrors that of primary appendicitis but is often associated with delayed diagnosis.

Several gaps remain in the current literature, including the absence of a standardized definition for high-risk stump length, limited prospective data on incidence in modern laparoscopic practice, and insufficient evidence regarding the outcomes of conservative management. Additionally, there are no established clinical guidelines or decision-making algorithms for evaluating recurrent RLQ pain in post-appendectomy patients with inconclusive imaging findings. A recurring limitation across the literature is the incomplete reporting of important variables such as stump length, the exact time interval since initial surgery, and the index operative approach, which further underscores the need for standardized reporting in future studies.

These findings emphasize the importance of prevention during the index surgery and the central role of computed tomography in diagnosis. Improved documentation and standardized reporting of stump characteristics and operative details are essential to facilitate future research and guideline development.

## Conclusions

Stump appendicitis is an uncommon but clinically significant complication following appendectomy. When imaging identifies an inflamed residual stump, completion appendectomy remains the definitive treatment in most cases. However, conservative management with antibiotics and/or percutaneous drainage may be appropriate in selected patients, particularly in the setting of complicated disease or when immediate surgery is not feasible. In patients with recurrent RLQ pain after appendectomy and no other identified cause, stump appendicitis should remain an important differential diagnosis. Prevention at the index operation, including clear identification of the appendiceal base and minimization of stump length, is critical. Future efforts should focus on standardized reporting, prospective data collection, and the clinical validation and implementation of proposed diagnostic and management algorithms to guide care in patients with ambiguous imaging and recurrent symptoms.
